# New insights into the pathogenesis and management of rheumatoid arthritis

**DOI:** 10.1002/cdt3.43

**Published:** 2022-09-21

**Authors:** Shangyi Jin, Jiuliang Zhao, Mengtao Li, Xiaofeng Zeng

**Affiliations:** ^1^ Department of Rheumatology and Clinical Immunology, Chinese Academy of Medical Sciences & Peking Union Medical College; National Clinical Research Center for Dermatologic and Immunologic Diseases (NCRC‐DID), Ministry of Science & Technology; State Key Laboratory of Complex Severe and Rare Diseases, Peking Union Medical College Hospital (PUMCH); Key Laboratory of Rheumatology and Clinical Immunology Ministry of Education Beijing China

**Keywords:** comorbidity, drug safety, JAK inhibitor, rheumatoid arthritis, single‐cell sequencing

## Abstract

Over the past few decades, understanding of the pathogenesis of rheumatoid arthritis (RA) has improved substantially. Insights into the cellular and molecular mechanisms involved in RA have enabled the discovery of new therapeutic targets and led to the development of biologics and targeted synthetic disease‐modifying antirheumatic drugs. In parallel with the improvement in therapies, the evolution of strategies in the management of RA has also contributed considerably to better outcomes in patients. Major changes include the development of disease activity measures, formulation of the treat‐to‐target principles as well as increased attention to comorbidities. The presence of comorbidities such as cardiovascular diseases may increase the mortality of RA patients, affect their treatment strategies and result in worse outcomes. Therefore, prevention and management of certain high‐risk comorbidities have become increasingly important in the long‐term treatment of RA. In this study, we summarized new insights into the pathogenesis and management of rheumatoid arthritis and associated comorbidities, with a special focus on the 2021 update of the American College of Rheumatology (ACR) guideline for RA and key reports presented at the 2021 ACR convergence.

## Key points

- The 2021 American College of Rheumatology guideline for rheumatoid arthritis (RA) provided updated recommendations on the use of disease‐modifying antirheumatic drugs, with a special focus on patients with certain high‐risk comorbidities, and reflected changes in the treatment of RA.
- Single‐cell sequencing, a novel technique capable of revealing the genomes and transcriptomes of individual cells, has been applied to reveal the underlying pathogenesis of RA.
- Risk evaluation and management of key comorbidities in RA, such as cardiovascular diseases, osteoporosis, malignancies, and infections have gained increasing attention in the research of RA.
- Selective JAK1 inhibitors showed promising results in both efficacy and safety in new reports from clinical trials. Further research is required to judge their optimal position in the treatment of RA.

## Graphical abstract

In this study, we summarized new insights into the pathogenesis and management of rheumatoid arthritis and associated comorbidities, with a special focus on the 2021 update of the American College of Rheumatology (ACR) guideline for rheumatoid arthritis and key reports presented at the 2021 ACR convergence.

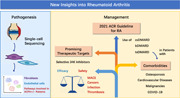

## 1 INTRODUCTION

Rheumatoid arthritis (RA) is a common autoimmune disease characterized by chronic symmetrical polyarthritis and various extra‐articular manifestations.^1^ With substantial advances in the research of the pathogenesis of RA, our understanding of the disease has improved. Based on these new insights, a variety of new therapies have been developed, which showed promising results in clinical trials. ACR convergence, the annual meeting of the American College of Rheumatology (ACR), is the leading forum for physicians and scientists all around the world who treat or research rheumatic diseases.^2^ In the year 2021, the ACR convergence was held virtually from November 5 to 9. More than 14,000 rheumatology professionals from over 110 countries participated and shared the most relevant and cutting‐edge information in this area. In the present review, mainly based on the studies reported in the 2021 ACR convergence, we summarized new insights into the pathogenesis and management of RA in the past few years.

## 2 2021 ACR GUIDELINE REFLECTED CHANGES IN RA TREATMENT

In the year 2021, ACR released an updated guideline for the management of RA.^3^ This update provided recommendations on several important topics, including the use of conventional synthetic disease‐modifying antirheumatic drugs (csDMARDs), biologic DMARDs (bDMARDs), targeted synthetic DMARDs (tsDMARDs), and glucocorticoids (GC) in the treatment of RA, with a special focus on patients with certain high‐risk comorbidities.

The 2021 ACR guideline no longer categorizes patients as having early RA or established RA. For DMARD‐naïve patients with moderate‐to‐high disease activity, methotrexate (MTX) remains to be the first choice for initial therapy. To ensure the efficacy of MTX, the 2021 guideline also gives specific recommendations on the dose and administration of MTX. Unlike the 2019 EULAR recommendations,^4^ the 2021 ACR guideline is more cautious with the use of GCs. Though short‐term GCs are commonly used as bridging therapy before csDMARDs exhibit their efficacy, the ACR guideline emphasized that GCs should not be systematically prescribed. In DMARD‐naïve patients with low disease activity, based on the concerns about safety, hydroxyquinoline (HCQ) is recommended. As for the modification of treatment, a treat‐to‐target approach is still strongly recommended over usual care in the 2021 guideline.^5^ For patients with inadequate response to initial treatment with csDMARD monotherapy, the guideline recommended the addition of bDMARDs or tsDMARDs over combinations of csDMARDs.

The presence of comorbidities may increase patients' risk to develop adverse effects from certain therapies. Therefore, comorbidities may have significant impacts on treatment strategies. Since the update in 2015,^6^ ACR guideline provides recommendations regarding the treatment of patients with certain comorbidities. The 2021 guideline continuously focused on these special populations. Recommendations have been updated based on new evidence (Table 1).

**Table 1:** Major updates in the 2021 American College of Rheumatology (ACR) guideline regarding treatment in rheumatoid arthritis (RA)

	2015 update	2021 update
1. Initiation of DMARDs	1. Early RA: Low/*Moderate‐to‐high disease activity*: Use DMARD monotherapy (MTX preferred) 2. Established RA: Low/*Moderate‐to‐high disease activity*: Use DMARD monotherapy (MTX preferred)	1. Low disease activity: *Use HCQ over other csDMARDs* 2. Moderate to high disease activity: Use MTX monotherapy over other csDMARD monotherapy, b/tsDMARDs, and combination therapies *Initiation of DMARDs without GCs over with GCs*
2. Modification of treatment after failure	Use a combination of csDMARDs or TNFi or non‐TNFi biologics	Addition of bDMARDs or tsDMARDs over combinations of csDMARDs
3. Administration of MTX	‐	*Oral over other ways of administration* *Initiation/titration of MTX to a weekly dose of at least 15 mg within 4 to 6 weeks*
4. Specific patient population	Heart Failure Hepatitis B Hepatitis C Malignancy Lymphoproliferative disorder Serious infection	Heart Failure Pulmonary disease Nonalcoholic fatty liver disease Lymphoproliferative disorder Hepatitis B Previous serious infection Nontuberculous mycobacterial lung disease

*Note*: Italicized text indicates conditional recommendations.

Abbreviations: DMARDs, disease‐modifying antirheumatic drugs; HCQ, hydroxyquinoline; MTX, methotrexate; RA, rheumatoid arthritis.

### 2.1 RA patients with heart failure

For patients with New York Heart Association (NYHA) class III or IV heart failure who respond inadequately to csDMARDs, the addition of non‐TNF inhibitor bDMARDs or tsDMARDs is recommended over TNF inhibitors (TNFi). If patients currently taking TNFi develop heart failure, they would be recommended to switch to non–TNF inhibitor bDMARDs or tsDMARDs. These recommendations were developed based on the risk of worsening heart failure observed in randomized controlled trials (RCTs) of TNFi in patients with heart failure without RA. The impacts of TNFi on heart failure in RA remain controversial.

### 2.2 Pulmonary diseases

Though pre‐existing lung disease has been reported to be a risk factor for MTX‐related pneumonitis, the overall risk of MTX contributing to the worsening of lung diseases is unclear. Therefore, as the anchor drug in the treatment of RA, MTX is still conditionally recommended over alternative DMARDs for the treatment of patients who have moderate‐to‐high disease activity and with clinically diagnosed mild and stable airway or parenchymal lung diseases, or incidental diseases detected on imaging.

### 2.3 Lymphoproliferative disorders

For patients with moderate‐to‐high disease activity and a previous lymphoproliferative disorder for which rituximab is an approved treatment, rituximab is conditionally recommended over other DMARDs. It is because that rituximab would not increase the risk of recurrence or worsening of these disorders.

### 2.4 Infections

Evaluation of past and present infections is important when choosing the optimal treatment strategies for patients with RA. In the 2021 update of the ACR guideline, recommendations for patients with the following infections are provided.

#### 2.4.1 Hepatitis B virus infection

For patients initiating any bDMARDs or tsDMARDs who are hepatitis B core antibody (HBcAb) and surface antigen (HBsAg) positive, prophylactic antiviral therapy is strongly recommended. For patients initiating rituximab, prophylactic antiviral therapy should be given if they are HBcAb positive, regardless of their HBsAg status. For patients initiating tsDMARDs or bDMARDs other than rituximab, frequent monitoring of viral load and liver enzyme could be enough if they are HBcAb positive and HBsAg negative.

#### 2.4.2 Previous serious infections

For patients with a serious infection within the previous 12 months, combinations of csDMARDs are recommended over bDMARDs or tsDMARDs, and the addition of DMARDs is recommended over the escalation of glucocorticoids.

#### 2.4.3 Nontuberculous mycobacterial (NTM) lung diseases

In patients who have NTM lung disease and moderate‐to‐high disease activity despite csDMARD monotherapy, the addition of csDMARDs is preferred over bDMARDs or tsDMARDs. If combination csDMARDs failed in the next stage, abatacept is recommended over other bDMARDs and tsDMARDs due to data extrapolated from studies on tuberculosis. In addition, because of the variability of NTM lung disease severity and response to treatment, patients with NTM lung disease should be closely comanaged with an infectious disease or pulmonary specialist.

Together with the recommendations on patients with hepatitis C virus infection and solid malignancies in the 2015 update, the ACR guideline gives rheumatologists evidence‐based guidance while making decisions for the treatment of these special patients. Some recommendations are conditional because of the low certainty evidence supporting them. Further research will be needed to formulate more solid recommendations.

## 3 SINGLE‐CELL SEQUENCING REVEALED THE UNDERLYING PATHOGENESIS OF RA

In addition to the rapid development of treatment strategies, advantages in basic science have also facilitated our understanding and control of this disease. Over the last 2 decades, the emergence of several novel techniques, such as multi‐omics approaches and artificial intelligence, has allowed us to explore the pathogenesis of RA in more depth.^7^, ^8^ Based on next‐generation sequencing technology, the rapidly developing single‐cell sequencing enables the profiling of genomes and transcriptomes of individual cells.^9^ Recently, single‐cell RNA sequencing (scRNA‐seq) has been applied in studies of RA, revealing some important aspects of its pathogenesis, including cell populations in synovial tissue, gene expression driving inflammation, and signaling pathways contributing to joint destruction.^10^, ^11^ Several studies applying this technique were introduced in the 2021 ACR convergence.

Regarding cells participating in the pathogenesis of synovitis, altered endothelial cell (EC) function and angiogenesis have been proven to play an important role. Edalat et al.^12^ created a comprehensive single‐cell atlas of synovial ECs in patients with inflammatory arthritis, which provides a reference data set for future EC subtype‐specific functional studies. As for inflammatory cells, by applying single‐cell RNA sequencing together with B and T cell repertoire sequencing in synovial tissue and matched peripheral blood, Meednu et al.^13^ revealed tissue‐specific clonal expansion and in situ selection of B and T cells in the synovium of RA patients. Regarding signaling pathways, Mueller et al.^14^ found that noncanonical Wnt signaling is a novel mechanism contributing to RA synovial fibroblast heterogeneity and inflammatory phenotype. Recently, Wu et al.^15^ revealed differential involvement of cellular and molecular pathways in the pathogenesis of seropositive and seronegative RA subtypes.

Single‐cell sequencing has also been applied to reveal the mechanisms of therapeutic response. For example, studying the transcriptome of T cells before and after antigen‐specific tolerizing immunotherapy helped Wehr et al.^16^ to evaluate its control of autoreactive T cells in RA. In another study published recently, after demonstrating the predictive ability of pretreatment T1IFN for patients' response to TNF inhibitors, scRNA‐seq was used further to examine the mechanism behind it.^17^

In the future, the application of scRNA‐seq in studies of RA may reveal the role of individual cell type and signaling pathway in the development and progression of the disease, and it may further facilitate the discovery of novel treatment targets. In addition to its contribution to pathogenesis, scRNA‐seq may also uncover the mechanism of inadequate response to certain medications, and further help in making treatment decisions.

## 4 MANAGING THE KEY COMORBIDITIES IN RA

Patients with RA are at higher risk of having a number of comorbidities, such as cardiovascular disease, fracture, and malignancy.^18^, ^19^, ^20^ The presence of comorbidities may increase the mortality of RA patients and affect their treatment strategies, resulting in worse outcomes.^21^ Therefore, management of comorbidities has become an increasingly important issue in the long‐term treatment of RA. In the 2021 ACR convergence, many studies regarding this topic were reported.

### 4.1 Cardiovascular diseases (CVDs)

CVDs remain to be the leading causes of death in patients with RA according to a newly reported cohort study in Australia.^22^ To improve the risk evaluation of CVDs in RA, Kuriya et al.^23^ estimated cardiac and lipid biomarkers in patients with RA and no diagnosis of CVDs. The results showed that abnormalities in biomarkers are already common in these patients, even in those with well‐controlled arthritis. As for cerebrovascular diseases, Vassilaki et al.^24^ found that patients with RA had more abnormalities in cerebrovascular imaging biomarkers, which may also have potential value for the prognostication of CVDs in RA. In addition to the impact of RA, medications are also associated with the risk of CVDs. Long‐term use of glucocorticoids, even in low dose, were shown to be related to increased risk for stroke and myocardial infarction.^25^ Tofacitinib, one of the rapidly developing JAK inhibitors (JAKi), was reported to be associated with an increased risk of major adverse cardiovascular events (MACEs) and cancers compared with a TNF inhibitor in patients who were 50 years of age or older and had at least one cardiovascular risk factor in a postauthorization safety trial (ORAL Surveillance).^26^ To examine this association, Khosrow‐Khavar et al.^27^ conducted a multi‐database study (STAR‐RA) with more than 10,000 patients treated with tofacitinib in real‐world settings. The study did not find evidence for an increased risk of CVDs in the overall study population. However, similar to the previous study, an increased risk did exist in patients with cardiovascular risk factors or a history of CVDs. Besides, in further analysis of the ORAL Surveillance, compared to patients treated by TNFi, patients receiving tofacitinib also showed an increased risk of developing venous thromboembolic events.^28^ Therefore, though there have been increasingly more studies proving the effectiveness of JAKi, more evidence is required to evaluate the safety of these drugs to be used in specific populations, such as patients with cardiovascular risk factors. In addition, in patients treated with JAKi, regularly planned monitoring during treatment should be performed for known risks.

### 4.2 Osteoporosis

Regarding the risk evaluation of incident fractures, high‐resolution peripheral quantitative computed tomography (HR‐pQCT), a novel noninvasive radiographic technique, has been shown to have predictive value in patients with rheumatologic diseases receiving glucocorticoids in a 5‐year longitudinal study.^29^ As for anti‐osteoporotic treatment, Adachi et al.^30^ compared denosumab with risedronate regarding their safety and efficacy in patients with RA and glucocorticoid‐induced osteoporosis. Treatment with denosumab showed a significantly larger improvement in BMD and a similar safety profile with risedronate.

### 4.3 Malignancies

A large cohort study in China investigated the risk of cancer in five major autoimmune diseases.^31^ The results showed that the standardized incidence ratio (SIR) for cancers in patients with RA was 3.99 (95% CI 2.40–3.65). RA patients had no significant difference in site‐specific SIRs, suggesting a wide distribution of cancer sites after 15 years since the diagnosis of RA. Regarding the association between drugs and cancers, JAKi was reported to result in a higher risk of cancers compared with TNFi in patients over 50 years old and with at least one CVD risk factor.^26^ Further analyses showed that older age and history of smoking were independent risk factors for cancers. Lung cancer was the most frequently reported cancer in patients treated with JAKi.^32^

### 4.4 Multimorbidity

Multimorbidity in RA was also reported in the 2021 ACR convergence. A cohort study in the United States showed that patients with RA had a higher prevalence of multimorbidity compared to non‐RA subjects,^33^ and a list of morbidities proposed by England et al. was advised to be used in studies on multimorbidity. Dykhoff et al.^34^ investigated gender differences in multimorbidity in RA. The results showed that multimorbidity was more frequently reported in women with RA. As for individual comorbidities, depression, hypothyroidism, fibromyalgia, asthma, gastroesophageal reflux disease, osteoporosis, and osteoarthritis were more common in women. Schieir et al.^35^ evaluated the impact of multimorbidity on disability over time and revealed that depression in women, and lung disease and hypertension in men were associated with increased disability in RA. Management of multimorbidity with additional focus on some key conditions may help improve the overall prognosis of RA.

### 4.5 COVID‐19

During the COVID‐19 pandemic, several concerns have emerged regarding the impact of the pandemic on patients with autoimmune diseases. Whether patients treated with immunosuppressants are more vulnerable to the pandemic? Whether vaccines are useful and safe for these patients? A recently published meta‐analysis indicated that compared to the general population, patients with rheumatic and musculoskeletal diseases have a higher risk of COVID‐19 infection and an increased mortality rate.^36^ In the 2021 ACR convergence, a study in the UK found that patients with inflammatory arthritis had a lower incidence of COVID‐19 but higher mortality compared to healthy controls.^37^ The relatively low incidence was attributed to patients' selfisolation at home (shielding), suggesting that shielding could be a helpful protective approach in RA patients. Medications of RA may also have an impact on the risk of COVID‐19 infection and outcomes. Compared with csDMARDs, the use of rituximab in patients with RA and COVID‐19 was associated with higher odds of hospitalizations and invasive ventilation.^38^ Regarding vaccination, a team in the Cleveland medical center demonstrated that the mRNA vaccine was safe in patients with RA and did not result in flares or side effects.^39^ However, another study showed that after vaccination, antibodies against COVID‐19 grew slower and reached lower titers in patients with RA, especially in those receiving abatacept or JAK inhibitors.^40^ Further research with a larger population is needed to evaluate the safety and effectiveness of the COVID‐19 vaccine in patients with RA.

## 5 PROMISING THERAPEUTIC TARGETS FOR THE TREATMENT OF RA

Among the rapidly developing treatment approaches for RA, tsDMARDs targeting Janus kinase–signal transducers and activators of transcription (JAK‐STAT) pathway have gained increasing attention in the past few years.^41^ With evidence supporting its efficacy in a variety of RA populations, tsDMARDs have gained a higher position in the treatment sequence in new updates of guidelines.^3^, ^4^ These small molecule drugs have targeted mechanisms of action, robust and rapid effects, and meanwhile maintain advantages of small molecule drugs, such as oral route of administration, easy to be stored, and relatively low cost compared with bDMARDs. However, despite the advantages of JAKi, evidence of its safety issue regarding the risk of infection, cancer, MACEs, and thrombosis has been reported and drug safety alerts have been released by FDA.^26^, ^42^ Though the mechanisms behind these adverse effects remain unclear, some of them may be attributed to the blockade of cytokines that use JAK–STAT for signaling. Therefore, the new generation JAKi inhibiting function of fewer cytokines with greater specificity is expected to have fewer adverse effects and improved safety.^43^

In the 2021 ACR convergence, new results from clinical trials of selective JAKi in the treatment of RA have been reported. Upadacitinib (UPA), a selective JAK1 inhibitor, has a series of clinical trials testing its efficacy and safety as monotherapy or in combination with csDMARDs at different stages in the treatment of RA. In DMARD‐naïve patients (SELECT‐EARLY), UPA monotherapy was compared with MTX monotherapy and showed more profound and rapid effects on biomarkers as well as sustained higher clinical response rate.^44^, ^45^ In patients with poor response to MTX monotherapy, the addition of UPA was compared with the addition of adalimumab in a phase 3 study (SELECT‐COMPARE).^46^ UPA‐treated patients achieved higher rates of remission or low disease activity, greater improvement in patient‐reported outcomes, and consistently higher sustained response rates in long‐term follow‐up. In patients with inadequate response to bDMARDs, over three‐quarters of patients on UPA 15 mg achieved low disease activity or remission, and the response was sustained in two‐thirds of them (SELECT‐BEYOND).^47^ in comparison with abatacept plus csDMARDs, UPA plus csDMARDs showed a better clinical response (SELECT‐CHOICE).^48^ Clinical trials of filgotinib, another JAK1 selective inhibitor, also showed promising results in efficacy.^49^

Regarding the safety of selective JAK1 inhibitors, though UPA was reported to be associated with higher rates of adverse events (AE), such as herpes zoster, neutropenia, lymphopenia, hepatic disorder, and elevation of creatine phosphokinase in these trials, severe AEs resulting in treatment discontinuation were infrequent.^45^, ^50^ Similar results were shown in the 3‐year long‐term extension (LTE) report of the SELECT‐COMPARE study published in Feb 2022.^51^ In the 3‐year follow‐up, despite the higher rates of the above AEs in UPA‐treated patients, rates of several relatively severe AEs were generally comparable between UPA and adalimumab, including AEs leading to discontinuation, serious infections, and serious AEs, malignancies, major adverse cardiac events, venous thromboembolism, and deaths. These promising results continuously support a favorable benefit: a risk profile for UPA in the treatment of RA. Future research investigating the long‐time safety of selective JAKi in large populations needs to be conducted to judge their overall efficacy and safety profiles, as well as their position in the treatment of RA.

## 6 CONCLUSIONS

In the past few decades, the rapid growth of research in RA has given us new insights into this common autoimmune disease. Development in basic science based on state‐of‐the‐art technologies, such as multi‐omics study and artificial intelligence, has improved our understanding of the pathogenesis of RA and its comorbidities, as well as the mechanisms of the action of drugs. On the basis of these novel findings, new therapies have been developed and examined, and our understanding of conventional therapies has also been improved. Meanwhile, we were enabled to build a comprehensive management system for RA, including prevention, early diagnosis, risk stratification, treat‐to‐target strategy, as well as management of comorbidities. The latest updates of guidelines and recommendations reflect these improvements, aiming at better care for RA patients worldwide. Several key questions, such as the comparative effectiveness and safety between the novel and conventional therapies, potential ways to predict response to certain therapies, as well as treatment strategies in patients with other comorbidities (e.g., solid malignancies), remain to be answered by future research.

## AUTHOR CONTRIBUTIONS

This review was designed by Shangyi Jin and Jiuliang Zhao. The manuscript was drafted by Shangyi Jin and revised by Jiuliang Zhao. Mengtao Li and Xiaofeng Zeng supervised the study. All authors approved the final manuscript.

## CONFLICT OF INTEREST

The authors declare no conflict of interest.

## ETHICS STATEMENT

Not applicable.

## Data Availability

Not applicable.
